# *In vivo *proton magnetic resonance spectroscopy reveals region specific metabolic responses to SIV infection in the macaque brain

**DOI:** 10.1186/1471-2202-10-63

**Published:** 2009-06-22

**Authors:** Eva-Maria Ratai, Sarah J Pilkenton, Jane B Greco, Margaret R Lentz, Jeffrey P Bombardier, Katherine W Turk, Julian He, Chan-Gyu Joo, Vallent Lee, Susan Westmoreland, Elkan Halpern, Andrew A Lackner, R Gilberto González

**Affiliations:** 1 Neuroradiology Division, Department of Radiology and A.A. Martinos Center for Biomedical Imaging, Massachusetts General Hospital, Charlestown, Massachusetts, 02129, USA; 2 Harvard Medical School, Boston, Massachusetts, 02115, USA; 3 New England Primate Research Center, Southborough, Massachusetts, 01772, USA; 4 Institute for Technology Assessment, Department of Radiology, Massachusetts General Hospital, Charlestown, Massachusetts, 02129, USA; 5 Tulane National Primate Research Center, Tulane University Health Science Center, Covington, Louisiana, 70433, USA

## Abstract

**Background:**

*In vivo *proton magnetic resonance spectroscopy (^1^H-MRS) studies of HIV-infected humans have demonstrated significant metabolic abnormalities that vary by brain region, but the causes are poorly understood. Metabolic changes in the frontal cortex, basal ganglia and white matter in 18 SIV-infected macaques were investigated using MRS during the first month of infection.

**Results:**

Changes in the N-acetylaspartate (NAA), choline (Cho), *myo*-inositol (MI), creatine (Cr) and glutamine/glutamate (Glx) resonances were quantified both in absolute terms and relative to the creatine resonance. Most abnormalities were observed at the time of peak viremia, 2 weeks post infection (wpi). At that time point, significant decreases in NAA and NAA/Cr, reflecting neuronal injury, were observed only in the frontal cortex. Cr was significantly elevated only in the white matter. Changes in Cho and Cho/Cr were similar across the brain regions, increasing at 2 wpi, and falling below baseline levels at 4 wpi. MI and MI/Cr levels were increased across all brain regions.

**Conclusion:**

These data best support the hypothesis that different brain regions have variable intrinsic vulnerabilities to neuronal injury caused by the AIDS virus.

## Background

Despite the successes of antiretroviral therapies, the neurocognitive complications of AIDS infection (neuroAIDS) continue to be an important problem [1]. Interestingly, clinical, neuropathological and imaging studies have demonstrated that the virus variably affects different regions of the brain [2-4]. *In vivo *^1^H MRS studies of chronically HIV infected patients have disclosed varying metabolic abnormalities in different brain regions [5,6]. Some have reported that the most profound MRS changes occur in the basal ganglia and white matter [7,8] while others have reported significant changes in the frontal cortex of HIV patients, even in patients free of neurological symptoms [9].

The simian immunodeficiency virus (SIV)-infected rhesus macaque model is arguably the best animal model of neuroAIDS [10]. There are strong neuropathological similarities between HIV patients and SIV-infected macaques [11,12]. *In vivo *macaque brain ^1^H MR spectra are similar to humans and *post mortem *and *in vivo *MRS studies from SIV infected macaques have revealed metabolic abnormalities [13-15] similar to those observed in HIV-infected human brains by *in vivo *MRS [16,17]. A power calculation conducted by Greco et al. revealed that single voxel MRS at 1.5 Tesla was capable of detecting changes similar to those observed in human MRS studies for most metabolites using less than 10 animals [18].

Eight of the animals reported on in this study were allowed to progress until terminal AIDS or the 2 year endpoint of the study. Pathology of the harvested brains revealed that two out of the eight animals developed SIVE encephalitis (SIVE) as defined by the presence of perivascular infiltrates of macrophages and multinucleated giant cells [19]. These findings are consistent with previous reports that neuropathological changes are variable, with SIV encephalitis developing in 25% of infected macaques [20] similar to the incidence of encephalitis in HIV-infected humans. Furthermore, spectroscopy on these animals revealed that an elevation of basal ganglia Cho/Cr at four weeks post-inoculation is predictive for the development of SIV encephalitis [19].

The objective of our study was to evaluate macaque brain regional variations during acute SIV infection. During the first month of SIV infection, very rapid changes in macaque blood viral load are observed [21] much like its human counterpart. We previously reported that profound metabolic changes occur in the frontal cortex of macaques in the first weeks after SIV infection [15]. To help understand brain region specific variations in HIV/AIDS, we evaluated the neurochemical changes observed by *in vivo *^1^H MRS in the basal ganglia and white matter, and compared them to the changes seen in the frontal cortex in 18 SIV-infected rhesus macaques. This study aims to reveal which brain regions are more susceptible to gliosis and neuronal injury during the primary phase of infection.

## Results

**Figure 1:**
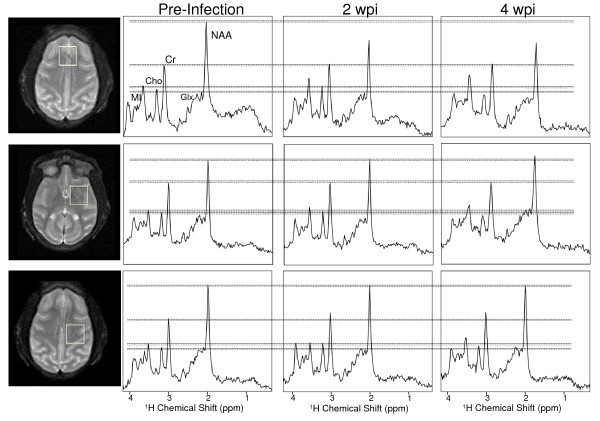
**Voxel locations (left) and corresponding representative spectra are shown for the frontal cortex (top), basal ganglia (middle), and white matter of the centrum semiovale (bottom)**. The major metabolites are labeled on the spectra, as well as dashed lines corresponding to pre-infection levels. Spectra are from a single representative animal, are not baseline corrected or apodized. Top: Frontal cortex MR spectra of a representative monkey before infection, at 2 and 4 wpi. Spectra are scaled to the creatine resonance because Cr levels did not change in frontal cortex during acute SIV infection. It can be seen from the spectra that NAA is decreased at 2, Cho is decreased at 4 wpi, and MI is increased at 2 and 4 wpi. (Due to the difficulty in finding spectra from a single animal that are representative of the group data in all aspects, we would like to note that the decrease in NAA at 4 wpi is not representative of the unchanged NAA concentration reflected in the group data). Middle: Basal ganglia MR spectra are scaled to the Cr peak as the Cr concentration did not change. No significant changes in NAA were observed. A decrease in Cho is evident by 4 wpi. Finally, an increase in MI can be observed from at 2 and 4 wpi. Bottom: White Matter MR spectra of a the same representative monkey before infection, at 2 and 4 wpi. Spectra are scaled to the NAA resonance because NAA levels did not change in white matter semiovale during acute SIV infection. It can be seen from the spectra that Cr is increased at 2 wpi, and Cho is increased and then decreased to baseline levels at 4 wpi. An increase in MI can also be detected.

A total of 18 rhesus macaques were infected with SIV, and imaged with *in vivo *^1^H MRS at multiple time points including pre-infection and up to 4 weeks after infection. ^1^H MR spectra were separately acquired from 3 specific brain regions, frontal cortex, basal ganglia, and centrum semiovale, as illustrated in Figure 1. ^1^H MR spectra with short echo time (TE = 35 ms) are characterized by resonances primarily arising from NAA, MI, Cr, Cho, and Glx (Figure 1). Peak viremia was observed at ~11–12 days pi (dpi) with a mean plasma level of 5.8 × 10^7 ^copy eq./mL. At 4 wpi, the mean plasma viral load was 4.0 × 10^6 ^copy eq./mL

A multivariate analysis of variance (MANOVA) was performed for all metabolites across all time points and brain regions, and highly significant differences were found (P < 0.0001). Further analyses isolated temporal (P < 0.0001) and regional differences (P = 0.007). Below, we detail the temporal metabolic changes during the first 4 weeks of SIV infection, and the metabolic changes that occur in different brain regions.

### Temporal changes in metabolite concentrations during acute SIV infection

**Figure 2:**
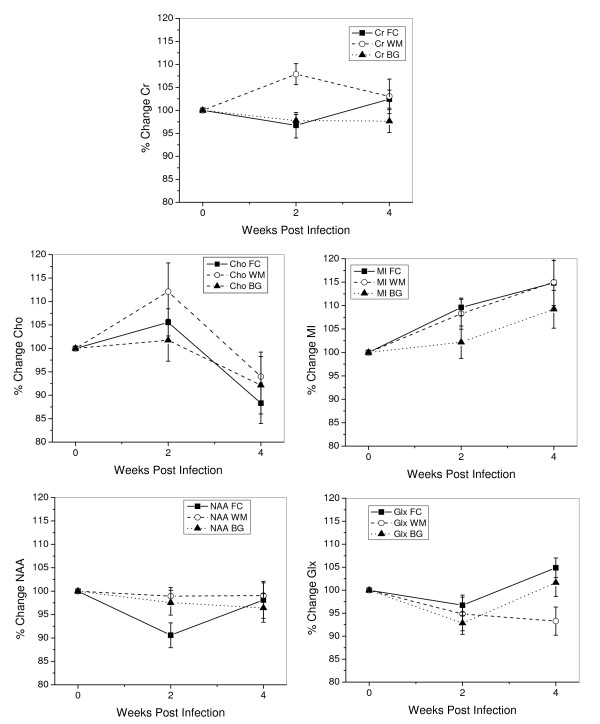
**Temporal spectroscopic changes observed in the normalized absolute concentrations of Cr, Cho, MI, NAA, and Glx in SIV-infected rhesus macaques measured in the frontal cortex (FC), white matter (WM) and basal ganglia (BG)**. Statistics are given in Table 1.

The normalized changes in the mean concentrations of NAA, Cr, Cho, MI, and Glx after infection in each brain region are graphically displayed in Figure 2. Metabolite concentrations (average levels ± SEM) and the results of the statistical analyses are listed in Table 1. P-values were obtained using repeated measures analysis of variance (RM ANOVA) on 12 animals measured before infection and at 2 and 4 wpi. If significant by RM ANOVA, Holm's t-tests were used to isolate differences between the time points. Additionally, paired t-tests were performed on all 18 animals scanned before infection and 2 wpi. NS indicated that ANOVA or t-tests were not significant. The most significant changes were observed in the frontal cortex and were consistent with previous studies [15]. NAA is significantly decreased at 2 wpi but is not different from baseline at 4 weeks. Cho shows a trend towards increase at 2 wpi (P = 0.077) but falls below baseline levels at 4 wpi. MI increases at 2 wpi and remains elevated. Cr concentration did not change over time in the frontal cortex. Thus, changes in metabolite concentration and metabolite ratios with respect to Cr are similar. No change in frontal Glx was identified.

**Table 1:** Average levels ± SEM of measured absolute metabolites in institutional units for NAA, Glx, Cr, Cho and MI in SIV infected macaques analyzed by *in vivo *MR spectroscopy

		**Frontal Cortex**	**White Matter**	**Basal Ganglia**
**NAA**	Uninfected	6.86 ± 0.19	7.94 ± 0.24	7.38 ± 0.15
	2 wpi	6.26 ± 0.15	7.82 ± 0.13	7.17 ± 0.16
	4 wpi	6.83 ± 0.79	7.92 ± 0.65	7.22 ± 0.68
	**RM ANOVA**	P = 0.025	NS	NS
	Holm't t-test pre – 2 wpi.	P = 0.011		
	Holm's t-test pre – 4 wpi	NS		
	Holm's t-test 2 wpi – 4 wpi	P = 0.035		
	**Paired t-test pre – 2 wpi**	P = 0.021	NS	NS
**Glx**	Uninfected	12.30 ± 0.26	11.71 ± 0.32	11.96 ± 0.23
	2 wpi	11.81 ± 0.29	11.14 ± 0.38	11.07 ± 0.29
	4 wpi	12.80 ± 0.29	11.26 ± 0.31	12.03 ± 0.30
	**RM ANOVA**	NS	NS	NS
	Holm't t-test pre – 2 wpi			
	Holm's t-test pre – 4 wpi			
	Holm's t-test 2 wpi – 4 wpi			
	**Paired t-test pre – 2 wpi**	NS	NS	P = 0.052
**Cr**	Uninfected	5.60 ± 0.09	5.76 ± 0.14	6.65 ± 0.10
	2 wpi	5.450 ± 0.13	6.15 ± 0.13	6.49 ± 0.08
	4 wpi	5.78 ± 0.10	6.01 ± 0.18	6.53 ± 0.14
	**RM ANOVA**	NS	P = 0.025	NS
	Holm't t-test pre – 2 wpi		P = 0.007	
	Holm's t-test pre – 4 wpi		NS	
	Holm's t-test 2 wpi – 4 wpi		NS	
	**Paired t-test pre – 2 wpi**	NS	P = 0.003	NS
**Cho**	Uninfected	1.014 ± 0.03	0.9754 ± 0.04	1.18 ± 0.03
	2 wpi	1.066 ± 0.025	1.078 ± 0.05	1.20 ± 0.04
	4 wpi	0.91 ± 0.04	0.9334 ± 0.04	1.10 ± 0.06
	**RM ANOVA**	P < 0.0001	P = 0.007	NS
	Holm't t-test pre – 2 wpi	P = 0.077	P = 0.008	
	Holm's t-test pre – 4 wpi	P = 0.016	NS	
	Holm's t-test 2 wpi – 4 wpi	P < 0.0001	P = 0.004	
	**Paired t-test pre – 2 wpi**	NS	NS	NS
**MI**	Uninfected	5.25 ± 0.14	4.72 ± 0.18	5.06 ± 0.12
	2 wpi	5.81 ± 0.08	5.15 ± 0.13	5.21 ± 0.15
	4 wpi	5.98 ± 0.23	5.45 ± 0.23	5.45 ± 0.18
	**RM ANOVA**	P = 0.048	P = 0.033	NS
	Holm't t-test pre – 2 wpi	P = 0.080	P = 0.051	
	Holm's t-test pre – 4 wpi	P = 0.018	P = 0.013	
	Holm's t-test 2 wpi – 4 wpi	NS	NS	
	**Paired t-test pre – 2 wpi**	P = 0.003	P = 0.029	NS

P-values were obtained using repeated measures analysis of variance (RM ANOVA) on 12 animals measured before infection and at 2 and 4 wpi. If significant by RM ANOVA, Holm's t-tests were used to isolate differences between the time points. Additionally, paired t-tests were performed on all 18 animals scanned before infection and 2 wpi. NS indicated that ANOVA or t-tests were not significant.

Interestingly, we found a significant increase in the Cr concentration at 2 wpi in the white matter of the centrum semiovale. Similar to the frontal cortex, white matter Cho increases significantly at the time of peak viremia at 2 wpi and decreases to a level below baseline at 4 weeks. Likewise, MI increases at 2 weeks and stays elevated at 4 weeks. However, NAA concentrations in the centrum semiovale show no significant differences during the first month of infection. A significant decrease in the white matter NAA/Cr (7.5% decline, P = 0.022) at 2 weeks is explained by an increase in Cr at that time point. Again, no significant Glx changes could be detected.

In the basal ganglia, absolute metabolite concentration changes over time were not statistically significant. However, while the Cho concentration did not change significantly at any time point after infection, Cho/Cr ratio did show a significant decline of 8.9% (P = 0.011) between 2 and 4 wpi. Inspection of the absolute concentration values of these 2 metabolites suggests that the change in the ratio is driven primarily by a decline in Cho, and the lower variances observed in ratios accounts for the observation of the statistically significant decline. Notably, a decline in Cho/Cr between 2 and 4 wpi was also observed in the frontal cortex (19.6% decline, P < 0.0001) and the white matter (10.5% decline, P = 0.019). Basal ganglia NAA and Glx changes after infection were not significant. If present, they are within the experimental error of the measurements.

### Correlations between metabolite concentration changes in different regions

To determine whether metabolic markers, NAA, Cho, MI, Cr and Glx levels change in a similar fashion during acute SIV infection in the frontal cortex, white matter and basal ganglia, Spearman Rank correlation analyses were undertaken. Table 2 summarizes the correlations between metabolite concentration changes in different regions. The rationale for correlating the changes of metabolites in different brain regions was to determine if the metabolic responses were uniform throughout the brain or if some regions are affected to a greater extent. We interpret positive correlations to mean that the responses to infection are similar in different brain regions, and that the responses are different when they do not correlate.

**Table 2:** Spearman Rank correlation analyses of metabolite concentrations between different brain regions of the SIV infected macaques.

	***NAA***		***Glx***		***Cr***		***Cho***		***MI***	
**Spearman Rank**	**R_s_**	**p**	**R_s_**	**P**	**R_s_**	**p**	**R_s_**	**p**	**R_s_**	**p**
**FC – WM**		NS	***0.32***	***0.088***		NS	**0.52**	**0.0032**		NS
**FC – BG**		NS	**0.52**	**0.0034**		NS	***0.36***	***0.052***	**0.37**	**0.043**
**WM – BG**	**0.50**	**0.0049**		NS		NS	**0.54**	**0.0021**		NS

For this purpose the percent change from baseline were calculated for each individual monkey for all time points. Significant P-values are noted in bold. P-values with a trend towards significance (P < 0.1) are noted italics and non-significant differences are indicated by NS. However, it must be noted that when the correlations are subjected to Bonferroni correction, a P-value of < 0.0033 is required for significance.

No significant correlations between NAA concentration changes in the frontal cortex and white matter, and between frontal cortex and basal ganglia NAA were found. While no significant temporal changes in NAA were found in the basal ganglia or white matter, Spearman Rank analysis revealed a significant association between white matter and basal ganglia changes (P = 0.0049, R_s _= 0.50). For the choline changes, Spearman Rank analyses revealed correlations between all three brain regions (frontal cortex vs. white matter P = 0.0032, R_s _= 0.52; frontal cortex vs. basal ganglia P = 0.052, R_s _= 0.36 and white matter vs. basal ganglia P = 0.0021, R_s _= 0.54). MI changes were correlated between frontal cortex and basal ganglia (P = 0.043, R_s _= 0.37), but not between white matter and the other two regions. There was no significant correlation between Cr concentration changes in the three brain regions. Finally, Glx changes were correlated between frontal cortex and basal ganglia (P = 0.0034, R_s _= 0.05), but, no correlation was found between white matter and basal ganglia. A trend was found between Glx changes in the frontal cortex and white matter.

### Correlations between metabolite concentration changes and plasma viral loads

We also investigated correlations between plasma viral load and metabolite concentration changes in the 3 volumes of interest (VOIs). Viral load data was obtained from 8 animals at 2 and/or 4 wpi for a total of 10 data points. Using Spearman Rank, we found a significant correlation between the change in the Cho/Cr ratio in the frontal cortex and change in the plasma viral loads at 2 and 4 weeks combined (R_s _= 0.67, P = 0.035). In addition, we found a correlation between plasma viral loads and Cho/Cr changes in the basal ganglia (R_s _= 0.70, P = 0.009) and Cho changes in BG (R_s _= 0.77, P = 0.02). There was no relationship between WM changes and plasma viral loads. Furthermore, there was no relationship between any of the other metabolite concentrations/ratios and viral loads. However, due to the small sample size, we consider these analyses highly preliminary.

## Discussion

A long-standing observation in patients infected with HIV is that different parts of the brain appear to be variably affected by the virus. Evidence for variable response to the virus by different brain regions has come from clinical, pathological and imaging studies. To help gain a better understanding of this phenomenon, we used *in vivo *^1^H MR spectroscopy to probe how different regions of the macaque brain respond metabolically during the first month of SIV infection, a time point not normally accessible by clinical studies. At the time of peak viremia, a transient decline in the NAA resonance, reflecting temporary neuronal injury, was only detected in the frontal cortex. On the other hand, changes in Cho and MI were similar across brain regions. The Cr resonance appeared stable in all regions and times with the exception of white matter where a transient increase was observed at peak viremia.

### Evidence of neuronal injury is detected only in the frontal cortex

A decrease in NAA is only observed in the frontal cortex at 2 wpi, the time of peak viremia. NAA is almost exclusively located in neurons, and a decrease in NAA reflects neuronal loss [22,23] or reversible neuronal injury [24,25]. Lentz et al. showed that encephalitis severity correlates with decreased NAA/Cr levels in the frontal cortex in the macaque model of neuroAIDS [26,27]. With the reduction of SIV plasma viral load by 1–2 orders of magnitude at 4 wpi, NAA levels are also no longer abnormal, suggesting that partial immunologic control of the virus is sufficient to result in neuronal recovery. The selective neuronal vulnerability of frontal cortex to SIV infection is further supported by the observation of significant decreases in synaptophysin (SYN) in the frontal cortex but not in the basal ganglia (unpublished results). SYN is an integral membrane glycoprotein in presynaptic vesicles. Its decline indicates a disruption in synaptic transmission and it is therefore a sensitive marker of neuronal dysfunction. We have previously shown that SYN is highly correlated to *in vivo *NAA/Cr measurements in this same SIV/macaque model of neuroAIDS [24].

### Choline changes in the same manner in the brain regions

The Cho levels appear to change in a similar fashion in all brain regions after SIV infection. This implies that the changes are global, and not specific to any particular brain region. MI increased significantly in the FC and WM, and MI changes correlated between frontal cortex and basal ganglia (P = 0.043, R_s _= 0.37), but not between white matter and the other two regions. It must be noted that when the correlations are subjected to Bonferroni correction, the MI correlations lose significance, however, the choline correlations between the regions remain significant after Bonferroni correction. At the time of peak viremia, Cho levels are elevated, and they decrease to baseline levels or lower with a reduction of plasma viral load at 4 wpi. An increase in Cho may reflect altered membrane metabolism [28], and may be associated with cerebral inflammation. Its elevation is believed to reflect cellular responses, including cell proliferation, recruitment, microgliosis, and/or astrocytosis in neuroAIDS [29,30]. Evidence of the association with astrocytosis is given by our observations of elevated GFAP at the same time point in this model [31].

In a previous report [15] we reported that Cho levels in the frontal cortex decreased to levels significantly below baseline by 4 wpi. In extending our analyses to other regions we find that at 4 wpi Cho levels are below baseline in the white matter and basal ganglia, but these are not significant. However, significant declines in Cho were observed in all 3 regions between 2 and 4 wpi, a period when viral loads are substantially reduced. The best interpretation of this observation is that it is a manifestation of brain recovery and healing.

Cerebral MI increased almost linearly after SIV infection. MI is located primarily in glia; thus, elevation of MI is considered to reflect increased glial cell activity [32]. An elevation of MI has been commonly reported in studies of chronically infected HIV patients [9,16]. The increases in MI were not as highly correlated between the brain regions, but they appeared to increase in all 3 regions almost linearly up to 4 wpi. We have previously reported that there is a complex relationship between astrocytosis and MI, as well as between astrocytosis and Cho [31]. The data reported here on Cho and MI continue to support a complex relationship among these measurements that occur when the animal is infected. Further studies are necessary to elucidate the relationships.

### Creatine increases during acute SIV infection in white matter

At the time of peak viremia, Cr concentrations were significantly increased in the white matter of the centrum semiovale. Changes in total Cr (phosphocreatine and creatine) are associated with altered energy metabolism. Elevated Cr levels have been reported in the frontal white matter of chronic HIV patients [33]. It is believed that the virus enters the brain through infected monocytes that later differentiate into macrophages [34,35]. During this process of mononuclear cell infiltration, glial-cell activation and proliferation may manifest high tissue metabolism which would explain an increase in Cr. Energy change as a function of enhanced glial activation is supported by our finding that the greatest increases in MI and Cho are in the same regions as the highest increase in Cr (Figure 2). Our finding that creatine changes are not uniform across the brain regions is consistent with the study by Chang et al. [33] who reported creatine increases in the white matter but decreases in the basal ganglia in later stages of AIDS dementia complex.

### Comparison of HIV and SIV

Most HIV MRS studies are performed during the chronic stage of infection, often with subjects who are on antiretroviral therapy. Our macaque study focuses on acute SIV infection, a period that has not yet been studied by MRS in HIV infection. Acute SIV infection induces the most profound NAA changes in the frontal cortex, and more subtle and difficult to detect changes in the basal ganglia and white matter. We hypothesize that HIV in humans might produce similar responses during the primary infection as those described here. During primary infection, HIV and SIV proliferate to very high levels for a short time. As the immune system responds to the infection, plasma virus drops to lower levels. There may be recurrence of high viremia with the progressive weakening of the immune system by the virus. Anti-retroviral therapy will suppress viral production at this stage. It is only at the last stage, with the collapse of the immune system, that very high viremia is once again observed. There are very few reported MRS studies of people with early [17] or late high viremia [36,37]. It is possible that the difference in viral loads in patients is the most important factor responsible for differences MRS patterns that have been reported.

The macaque model has similar MR spectroscopic changes (diminished NAA, elevated Cho and MI) as in human neuroAIDS, suggesting that neurochemical and cellular responses to SIV and HIV are similar. However, the specific pattern of MRS changes in various brain regions of the acutely SIV-infected macaque appears different from that seen in humans. Both, single voxel MRS and magnetic resonance spectroscopic imaging (MRSI) studies performed before the advent of anti-retroviral therapy demonstrated profound declines in the levels of NAA in advanced cognitively impaired patients [29,38,39]. In the early stages of cognitive dysfunction, Cho/Cr and MI/Cr were shown to be elevated prior to changes in NAA [40].

More recently in the anti-retroviral era, *in vivo *^1^H MRS studies of HIV patients have consistently found metabolic abnormalities in different brain regions [5,6]. Some groups have reported that the most profound MRS changes occur in the basal ganglia and white matter [7,8]. Others have reported significant changes in the frontal cortex of HIV patients, even in patients free of neurological symptoms [9]. Additionally, recent high spatial resolution neuroimaging investigations using sophisticated analytic methods have found diminution in the thickness of the cortical mantle [41].

In this study we found region specific responses with respect to NAA but not choline. MRSI studies investigating the regional metabolic responses in HIV infected patients with HIV associated dementia were found to be quite variable. Some report evidence of uniform metabolic abnormalities (decreased NAA/Cr [39] and increased Cho/Cr) in various brain regions and conclude that abnormalities of cerebral metabolites in HIV-infected patients may be part of a diffuse process [42]. However, some MRSI studies observe NAA/Cr decreases [16] and Cho/Cr increases [43] in gray matter regions and not in white matter in patients with AIDS dementia complex. Additionally, Barker et al. reported that the largest reductions in NAA and increases in Cho were in the frontal lobe white matter and there were no changes in the cortical gray matter [38]. This great variability could be the result of many factors including: disease stage; the presence, type and duration of antiretroviral treatment; co-morbidities including drug abuse; and differences in imaging parameters such as pulse sequence, TE and TR.

We also investigated potential correlations between plasma viral load and metabolite concentration changes. We previously reported [15] a significant relationship between changes in Cho/Cr and plasma viral loads (R_s _= 0.79, P < 0.01) by Spearman Rank correlation analyses. Here, we found a correlation between plasma viral loads and Cho/Cr changes in the basal ganglia and Cho changes in BG. There was no relationship between WM Cho changes and plasma viral loads or between any of the other metabolite concentrations/ratios and viral loads. These preliminary findings are different from what has been reported for patients with HIV, however, our study focuses on the acute phase of infection, unlike human studies which involve chronically infected individuals. Among HIV+ patients higher plasma viral loads are typically associated with lower NAA [44] and higher MI [33]. Another study reported that CSF but not plasma viral load correlated with Cho/Cr in the white matter [45]. Again, there are many potential reasons for differences in correlations that have been reported between viral loads and MRS metabolites.

### Possible mechanisms of regional brain metabolic abnormalities

The leading theory of neuroAIDS pathogenesis is that infected/activated monocytes traffic into the CNS, where they differentiate into macrophages, and set off a cascade of events that ultimately result in neuronal injury [46]. One possibility that may explain regional variations in brain injury is that there are differences in the rate of monocyte trafficking into various brain regions. However, the similarity in the changes in Cho and MI observed in all regions suggests that the trafficking is globally similar. This is supported by the finding of similar astrocytic activation in frontal cortex and basal ganglia (unpublished results). However, while the evidence suggests that viral invasion is globally similar, neuronal injury is selective to the frontal cortex. Cortical neurons may be more susceptible to injury or neuronal protective/recovery mechanisms may be more effective in other regions.

The relationship of brain metabolic changes with respect to the level of virus in the blood is important. The greatest changes in NAA, Cho and Cr occur at the time of peak viremia followed by near normalization within 2 weeks. This sensitivity of the brain to events in the blood indicates a highly dynamic pathogenic process with the rapid transit of virally-infected monocytes into the brain and an equally rapid response by the brain to these events. The highly dynamic nature is further supported by the rapid normalization as plasma viral load decreases. Notably, this normalization occurs despite persistent virus in the blood, although not at peak levels. This observation suggests there may be a competition between pathogenic and protective mechanisms, and that a threshold level of viral load and trafficking, virally-infected monocytes must be exceeded for brain metabolic response to be manifested.

### Patient Management

Our results imply that the virus has a global effect on microgliosis and astrogliosis. However, only cortical regions, here the frontal cortex, are affected by neuronal injury, suggesting that some regions of the brain are more vulnerable to invasion by SIV or HIV infected macrophages. These results may support the use of a combination of antiretroviral therapy early to decrease influx of infected/activated monocytes/macrophages that lead to inflammation and neuroprotective drugs which pass the BBB in order to prevent neuronal injury in region of the brain in which the innate recovery mechanisms fail.

MR spectroscopy should be considered for use in clinical HIV patient care to monitor changes in metabolism indicative of neuronal dysfunction possibly predictive of cognitive dysfunction. Single voxel MRS [29,33,47] as well as magnetic resonance spectroscopic imaging (MRSI) [16,30,36,38,39,42,43,48,49] have been performed on patients with HIV dementia. Single voxel MRS has been commonly used since it is a shorter scan (for one voxel location) and requires less data processing. However, MRSI is a much more efficient technique to record MR spectroscopy data since it examines multiple regions in one data acquisition. Both techniques have shown significant changes in metabolite concentration in individuals with neuroAIDS and offer complementary information and should be used in conjunction as suggested by Sacktor [43] if time allows.

## Conclusion

In summary, during acute SIV infection, changes in Cho and MI were similar across brain regions while a significant decrease in NAA, a marker of neuronal injury, was found in the frontal cortex only. A potential mechanism for these observations is that trafficking of infected monocytes involves all brain regions producing cellular changes (astrocytosis, microgliosis) that are reflected in elevations in Cho and MI. However, the data suggest that there is variable regional susceptibility to neuronal injury. With respect to clinical HIV patient management, the data from this model suggests that while the virus has global effects such as astrogliosis, cortical regions may be more susceptible to neuronal injury and merit observation, perhaps with the inclusion of clinical MRS studies. Moreover, the use of neuroprotective agents may be particularly useful in the protection of cortical neuronal integrity. The SIV-infected macaque model monitored with ^1^H MRS is an excellent tool to further delineate the precise neuropathogenic mechanisms and to help assess the value of drugs that may used as adjunctive therapy whose purpose is the maintenance of cognitive normality.

## Methods

### Animals

The animals were housed according to the standards of the American Association for Accreditation of Laboratory Animal Care. Investigators adhered to the Guide for the Care and Use of Laboratory Animals of the Institute of Laboratory Animal Resources, National Research Council. The study was approved by the Massachusetts General Hospital Subcommittee on Research Animal Care and the Institutional Animal Care and Use Committee of Harvard University. Eighteen juvenile rhesus macaques (*Macaca mulatta*) of both sexes were included in the *in vivo *MRS study. All 18 animals had MR scans prior to infection with SIVmac251 (50 ng p27/kg) [50]. A second MR scan was obtained at 2 weeks post infection (wpi). Twelve of the eighteen animals underwent a third MR examination at 4 wpi. This imaging schedule was adopted because acutely infected macaques are unable to tolerate more frequent scanning. Animals were housed at the New England Regional Primate Research Center and transported to the Massachusetts General Hospital/A.A. Martinos Center for Biomedical Imaging. For imaging, the animals were tranquilized with ketamine or telazol and anesthetized with sodium pentobarbital. Six of these animals were sacrificed during the acute SIV infection for *postmortem *studies (*ex vivo *MR spectroscopy and quantitative neuropathologic analysis) the day after they underwent their last MR examination [24,31]. Eight of the remaining 12 animals were also included in the previously reported longitudinal *in vivo *^1^H MR spectroscopy study [19].

### MRI and MRS Studies

Both MRI and ^1^H MRS were performed using a standard GE linear extremity coil on a clinical 1.5 Tesla General Electric (Milwaukee, Wisconsin) Signa Scanner with an operating system of Horizon 8.3. First, a three-plane localizer was performed to position the monkey in the coil. In this manner, voxel placement was highly reproducible. A sagittal T1-weighted image sequence (TE/TR = 20/600) was utilized, followed by an axial dual echo pulse sequence (TE(1) = 30 ms; TE(2) = 80 ms; TR = 2500 ms) consisting of proton-weighted and T2-weighted images from which the ^1^H MRS voxels were prescribed. With the exception of a slightly smaller voxel size, the MRS protocol was identical to the one employed in multicenter human HIV studies [5]. MRS was performed on each animal in frontal cortical gray matter, white matter semiovale and putamen using a voxel size of 15 mm × 15 mm × 15 mm (3.4 cm^3^) (Figure 1). Data were acquired using an automated PROBE-P spectroscopy package [51], which consists of a PRESS sequence (TE = 35 ms, TR = 3000 ms, number of acquisitions = 128) [52] with CHESS water suppression [53].

All spectra were processed offline using the LCModel software package [54] to determine the quantities of the brain metabolites N-acetylaspartate and N-acetylaspartylglutamate (collectively referred to as NAA), choline-containing compounds (referred to as Cho), MI, creatine-containing compounds (referred to as Cr) and the so-called Glx concentration (while glutamate and glutamine represent the largest contributors to this peak area, we are aware that at 1.5T field strengths Glx will also include resonances from GABA, NAA, aspartate, and possibly succinate). Metabolite concentrations were estimated using the unsuppressed water signal from the same voxel, which served as the internal standard [55]. Absolute concentrations given by LCModel are normalizations of each resonance to the water peak, resulting in institutional units. Only if the tissue water concentration and T1 and T2 relaxation effects were estimated correctly, these units reflect a millimolar concentration. Metabolite ratios with respect to creatine were also calculated.

It is possible that alterations in metabolite transverse (T2) relaxation times are responsible for changes in metabolite resonance signals [56]. That is unlikely in the experiments reported here for several reasons. First, during the first month of SIV infection, we did not observe any signal changes on the anatomic T2 weighted images in these macaque brains suggesting little if any T2 effects. Additionally, some signal intensities increase at the same time that others decrease. If T2 changes were responsible, then some metabolites would be experiencing a prolongation in the transverse relaxation time while it would be shortened in others, a highly unlikely scenario. Moreover, we have previously reported high correlations between *in vivo *NAA measurements and *post mortem *tissue extract levels of this metabolite. The *ex vivo *measurements are made using a single pulse, long TR method thus eliminating T2 effects.

### Viral loads

After the MRI/MRS examination, blood samples were drawn from each animal to determine viral loads. Virion-associated SIV RNA in plasma was measured by using a real-time reverse transcription-PCR assay on an Applied Biosystems (Foster City, CA) Prism 7700 sequence detection system as described previously [57,58]. Results are averages of duplicate determinations.

### Statistical Methods

The preinfection values of each metabolite were different in the three brain regions. Thus for clarity, the data were normalized by setting the average preinfection metabolite concentration to 100 percent. Data taken at subsequent time points were normalized by multiplying by the same proportionality factor specific to brain region and metabolite. To test our hypothesis that all major cerebral metabolites are changing with time after SIV infection in a region-dependent manner, a multivariate analysis of variance (MANOVA) was performed with respect to metabolite, time post infection and brain region. Post hoc repeated measures analyses of variance (RM ANOVAs) for individual metabolites across time and region were carried out only if the MANOVA was significant (p < 0.05). MRS studies of uninfected macaques demonstrated that the standard deviation between scans of the same animal at different times was much smaller than the standard deviation between animals [18]. Statistical analyses of metabolite concentrations and metabolite ratios were performed using RM ANOVA on the data from the 12 animals that were scanned before infection, at 2 and 4 weeks after infection. If significant by RM ANOVA, Holm's t-tests, which correct for multiple comparisons, were used to isolate significant differences between time points.

For the 18 animals scanned at 0 and 2 wpi, paired t-tests were used to compare preinfection scans to the 2 week pi scans. A p-value of less than 0.05 was considered significant. To assess interrelationships between metabolites in the three brain regions, Spearman Rank correlation coefficients were used to assess potential associations between regions for each metabolic marker. Again, a P-value of less than 0.05 was considered to indicate a statistically significant correlation. However, it must be noted that when the correlations are subjected to Bonferroni correction (five metabolites and 3 regions), a P-value of < 0.0033 is required for significance. Additionally, potential relationships between plasma viral loads and brain metabolite changes at 2 and 4 weeks combined were assessed using Spearman Rank correlations. Finally, a power calculation was performed based on metabolite concentrations acquired from four animals that were scanned ≤ 4 times pre inoculation over a period of 2 years. We found the highest reproducibility in Cr > Glx > NAA > MI > Cho. The minimum detectable difference was between 5 and 16% for the metabolites with 80% power and a 5% significance level, in 18 animals.

## List of abbreviations used

NAA: N-acetylaspartate; Cr: Creatine; Cho: choline containing compounds; MI: *myo*-inositol; Glx: glutamate and glutamine; SIV: simian immunodeficiency virus; HIV: human immunodeficiency virus; CNS: central nervous system: MRS: magnetic resonance spectroscopy; MRSI: magnetic resonance spectroscopic imaging; wpi: weeks post infection, GFAP: glial fibrillary acidic protein; SYN: synaptophysin.

## Authors' contributions

EMR participated in the acquisition, statistical analysis and interpretation of the data and drafted the manuscript. SJP participated in the data analysis and interpretation of data and helped drafting the manuscript. JBG supervised and participated in the imaging experiments and analysis. MRL helped supervise and conduct imaging experiments, participated in image analysis and helped conduct statistical analysis. JPB participated in the statistical analysis and interpretation of the data and helped drafting the manuscript. KWT participated in the analysis of data. JH participated in animal care during imaging experiments, and participated in image analysis. CGJ participated in the analysis of data. VL helped drafting the manuscript. SVW participated on animal care and conducted pathologic evaluations. EH supervised and helped conduct statistical analysis. AAL participated in the design of the study, supervised animal care and pathologic evaluations. RGG conceived of the study, participated in its design and directed its execution. All authors read and approved the final manuscript.
